# Traditional Chinese medicine-derived monomers delay osteoarthritis progression by regulating mitochondrial homeostasis

**DOI:** 10.3389/fcell.2026.1845072

**Published:** 2026-05-20

**Authors:** Baohui Wang, Bo Dong

**Affiliations:** Traditional Chinese Medicine Pain Department, TCM Specialty Diagnosis and Treatment Center, Honghui Hospital, Xi’an Jiaotong University, Xi’an, China

**Keywords:** chondrocytes, mitochondrial homeostasis, mitophagy, osteoarthritis, TCM-derived monomers

## Abstract

Osteoarthritis is a common degenerative disease characterized by the degeneration of articular cartilage, which also affects the synovium, subchondral bone, and the joint microenvironment. Currently, clinical treatment remains focused primarily on pain relief and symptom improvement, with a lack of disease-modifying strategies capable of effectively slowing disease progression. In recent years, the role of mitochondrial homeostasis imbalance in the pathogenesis and progression of osteoarthritis has gradually gained attention. Mitochondria not only participate in the energy supply of chondrocytes but are also closely associated with oxidative stress, mitochondrial dynamics, mitochondrial autophagy, apoptosis, cellular senescence, and extracellular matrix metabolism. In the osteoarthritis microenvironment, inflammatory stimuli, abnormal mechanical loading, and age-related stress disrupt mitochondrial function, leading to reactive oxygen species accumulation, membrane potential decline, and impaired energy metabolism, which in turn promote chondrocyte dysfunction and joint degeneration. A growing body of research indicates that single compounds derived from traditional Chinese medicine can exert protective effects by regulating mitochondrial homeostasis, thereby alleviating oxidative stress, improving energy metabolism, maintaining mitochondrial function, promoting moderate mitochondrial autophagy, and inhibiting chondrocyte apoptosis and senescence. Ultimately, these actions reduce inflammatory responses and matrix degradation, thereby delaying the progression of osteoarthritis. This article reviews the pathological role of mitochondrial homeostasis imbalance in osteoarthritis, summarizes recent research progress on TCM-derived monomers, and outlines current challenges and future directions.

## 1 Introduction

Osteoarthritis (OA) is a common systemic joint disease characterized by the degeneration of articular cartilage, accompanied by synovial inflammation, subchondral bone remodeling, and impaired joint function. Current clinical treatment remains primarily focused on analgesia, anti-inflammation, and symptom management, with a lack of disease-modifying strategies capable of truly slowing structural progression. Therefore, elucidating its key pathogenic mechanisms and identifying new intervention targets is of great significance (Wang et al., 2020; Hwang and Kim, 2015; Kan et al., 2021). As understanding of chondrocyte metabolic abnormalities has advanced, mitochondria are no longer regarded simply as energy-supplying organelles, but as central hubs integrating ATP production, redox homeostasis, mechanostress responses, and cell fate regulation. Increasing evidence also suggests that disruption of mitochondrial quality control is involved in multiple key stages of OA pathogenesis and progression (Liu et al., 2022; Jiang et al., 2021; Wang et al., 2015). In the OA microenvironment, inflammatory stimuli, abnormal mechanical loading, and age-related stress can collectively induce mitochondrial homeostasis imbalance, manifested as impaired respiratory chain function, decreased membrane potential, excessive accumulation of ROS, and insufficient mitochondrial biogenesis. This, in turn, further amplifies the inflammatory response and promotes chondrocyte catabolism and extracellular matrix degradation (He et al., 2020; Vaamonde-García et al., 2012; Zhang et al., 2024).

Further studies have shown that abnormalities in mitochondrial-associated apoptotic pathways, autophagy, and mitophagy are closely associated with the progression of OA, suggesting that maintaining mitochondrial homeostasis is not only a key entry point for understanding the mechanisms of cartilage degeneration but may also represent a potential therapeutic approach for delaying the progression of OA (Takayama et al., 2009; Jin et al., 2022; Zhao et al., 2018). At the same time, an increasing number of single compounds derived from traditional Chinese medicine have been shown to exert protective effects by inhibiting inflammation and apoptosis, enhancing autophagy or mitophagy, restoring mitochondrial membrane potential, and alleviating chondrocyte damage. Among these, representative compounds such as quercetin, baicalin, and curcumin have demonstrated clear experimental evidence (Hu et al., 2019; Li et al., 2020; He and He, 2023). Accordingly, this review discusses the pathological role of mitochondrial homeostasis imbalance in OA, summarizes the mechanisms by which TCM-derived monomers regulate mitochondrial homeostasis, and outlines current challenges and future research directions.

## 2 The pathological role of mitochondrial homeostasis dysregulation in OA

Mitochondrial homeostasis dysregulation has been recognized as one of the key pathological mechanisms underlying the onset and progression of OA. Its significance lies not only in “energy deficiency” but also in its ability to simultaneously influence multiple critical processes, including oxidative stress, mechanical transduction, cell fate determination, and matrix metabolic balance (Blanco et al., 2011). In normal articular cartilage, although chondrocytes primarily rely on glycolysis for energy, mitochondria continue to play a role in ATP supplementation, calcium homeostasis, redox regulation, and stress signal integration. Therefore, the functional integrity of mitochondria is directly related to the phenotypic stability of chondrocytes and their ability to synthesize the extracellular matrix (Wu et al., 2024). Once the mitochondrial quality control network is compromised, the tolerance threshold of chondrocytes to inflammatory factors, aging stimuli, and mechanical loads decreases, ultimately leading to a gradual progression from compensatory imbalance to irreversible degeneration of articular cartilage (Fang et al., 2023).

**FIGURE 1:**
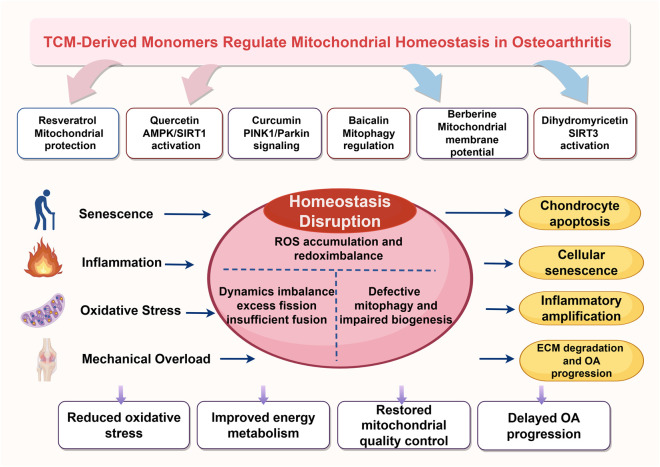
Schematic diagram illustrating the overall mechanism by which monomers derived from traditional Chinese medicine regulate mitochondrial homeostasis to slow the progression of osteoarthritis. Aging, inflammation, oxidative stress, and abnormal mechanical loading in the osteoarthritis microenvironment can disrupt mitochondrial homeostasis in chondrocytes, leading to reactive oxygen species accumulation, redox imbalance, metabolic dysfunction, impaired mitophagy, and reduced biogenesis. These changes promote chondrocyte apoptosis, senescence, inflammation, and extracellular matrix degradation, ultimately accelerating osteoarthritis progression. TCM-derived monomers, such as resveratrol, quercetin, curcumin, baicalin, berberine, and dihydroquercetin, may delay disease progression by improving mitochondrial function and quality control.

Among the manifestations of mitochondrial homeostasis imbalance, oxidative stress is one of the earliest and most self-amplifying events. In OA cartilage, mitochondrial respiratory chain dysfunction promotes persistent reactive oxygen species accumulation, while excessive mitochondrial reactive oxygen species further damage lipids, proteins, and mitochondrial DNA, thereby creating a vicious cycle of progressive mitochondrial injury (Chen L. et al., 2025). Concurrently, a decrease in mitochondrial membrane potential and reduced oxidative phosphorylation efficiency lead to insufficient ATP production, causing chondrocytes to experience significant energy supply deficits when faced with inflammatory stimuli or matrix repair demands, and further inducing a catabolic phenotype and suppression of cartilage matrix synthesis (Jiang et al., 2021; Wang et al., 2025). Furthermore, chondrocytes in OA exhibit reduced activity in mitochondrial biogenesis pathways, as represented by PGC-1α, SIRT1, and TFAM. This implies that not only do damaged mitochondria accumulate more readily, but the capacity for replenishment with new mitochondria is also simultaneously weakened, thereby causing energy metabolism disorders to become persistent and chronic (Dalmao-Fernández et al., 2021) (Figure 1).

In addition to oxidative stress and abnormalities in energy metabolism, mitochondrial homeostasis is another critical pathological factor in OA that cannot be overlooked. Under normal conditions, mitochondria maintain morphological renewal, stress adaptation, and functional differentiation through a dynamic balance of fission and fusion. However, in OA, abnormal mechanical loads and inflammatory stimuli can shift this balance toward excessive fission, leading to mitochondrial fragmentation, unstable membrane potential, and increased vulnerability to external stimuli (He et al., 2020; Xu et al., 2021). Concurrently, decreased expression of key molecules involved in maintaining mitochondrial homeostasis, such as SIRT3, further impairs mitochondrial antioxidant capacity, dynamic regulation, and deacetylation-mediated protective functions, making chondrocytes more susceptible to senescence and damage (Li et al., 2023). Notably, the disruption of mitochondrial homeostasis does not remain confined to the mitochondrial level; rather, it triggers mitochondria-dependent apoptosis through pathways such as Bax/Bcl-2 imbalance, cytochrome c release, and caspase cascade activation, thereby directly leading to a reduction in chondrocyte numbers and a decline in matrix maintenance capacity (Xu et al., 2020).

Mitochondrial autophagy is a central quality control mechanism for maintaining mitochondrial homeostasis. Its primary function is to identify and eliminate depolarized or damaged mitochondria, thereby preventing the continued accumulation of ROS and maintaining the renewal of the intracellular organelle network (Ansari et al., 2018)^.^ However, in the course of OA, mitochondrial autophagy is not simply a case of “the stronger, the better”; its protective or damaging effects exhibit distinct stage-specific and dose-dependent characteristics. For example, Pink1/Parkin-mediated mitochondrial autophagy has been found in some models to be associated with increased mitochondrial fragmentation and chondrocyte death, suggesting that abnormally activated or uncontrolled mitochondrial autophagy may also contribute to cartilage degeneration (Shin et al., 2019). On the other hand, recent studies have shown that moderately restoring or precisely enhancing mitochondrial autophagy can alleviate oxidative stress, improve mitochondrial quality, and alleviate the OA phenotype (Figure 1). For instance, α-ketoglutarate supplementation has been shown to mitigate OA damage by regulating mitochondrial autophagy and oxidative stress, indicating that the key lies not in simple enhancement or inhibition, but in restoring the dynamic balance between mitochondrial autophagy and mitochondrial function (Liu L. et al., 2023).

Furthermore, the reason why mitochondrial homeostasis imbalance continues to drive the progression of OA lies in the fact that it forms a mutually reinforcing pathological network with inflammatory responses, aging processes, and the remodeling of the joint microenvironment. Previous studies have shown that damaged mitochondria can enhance the sensitivity of chondrocytes to inflammatory factors such as IL-1β and TNF-α, and promote the upregulation of COX-2, iNOS, and various catabolic mediators, thereby causing the original low-grade inflammatory state to continuously self-amplify (Fang et al., 2023; Chen et al., 2023). Concurrently, improving mitochondrial autophagy and quality control often simultaneously alleviates cellular senescence, inflammatory amplification, and cartilage degeneration. For instance, FUNDC1/PFKP-associated mitochondrial autophagy, the HCAR2/AMPK/PINK1/Parkin axis, and OPTN-mediated mitochondrial autophagy have all been shown to be associated with chondroprotection and OA relief, which further demonstrates, from the opposite perspective, that the disruption of mitochondrial homeostasis serves as a critical hub linking inflammation, senescence, and structural damage (Zhuang et al., 2024; Zhang M. N. et al., 2025). Therefore, mitochondrial homeostasis imbalance is not merely a concomitant phenomenon in OA, but rather a pathological hub that runs throughout the disease course, integrating mechanical stress, metabolic dysfunction, inflammatory amplification, and dysregulation of cell fate. This also serves as the logical starting point for subsequent discussions on the value of interventions using single compounds derived from traditional Chinese medicine (Figure 1). Although this article primarily focuses on mitochondrial homeostasis abnormalities in chondrocytes, as a whole-joint disease, the pathological progression of osteoarthritis also involves various tissue-level changes, including synovial inflammation and subchondral bone remodeling. Previous studies have suggested that mitochondrial dysfunction may also contribute to these pathological processes; therefore, understanding its mechanisms of action from the perspective of the whole-joint microenvironment can help provide a more comprehensive understanding of the pathogenesis and progression of osteoarthritis.

## 3 Compounds derived from traditional Chinese medicine delay the progression of OA by regulating mitochondrial oxidative stress and energy metabolism

**FIGURE 2:**
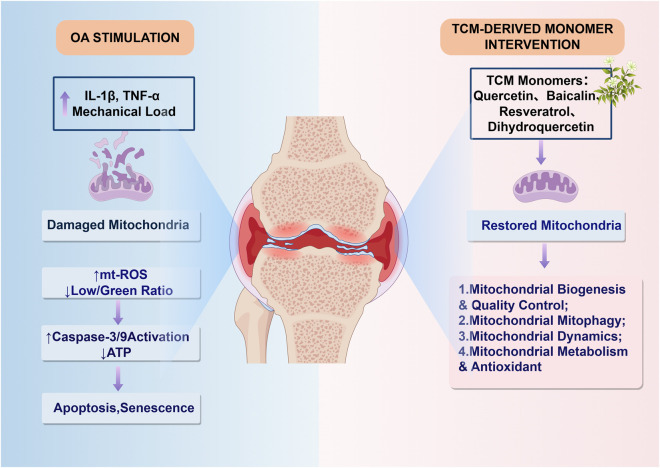
Mechanism of action of herbal-derived compounds in improving mitochondrial function and delaying the progression of osteoarthritis. OA-related stimuli, such as IL-1β, TNF-α, and abnormal mechanical loading, can cause mitochondrial damage, leading to the accumulation of mtROS, decreased ATP levels, and activation of caspase-3/9, which further promotes chondrocyte apoptosis and senescence. Compounds derived from traditional Chinese medicine can improve impaired mitochondrial function by enhancing mitochondrial biogenesis and quality control, regulating mitophagy and mitochondrial dynamic homeostasis, and strengthening mitochondrial metabolism and antioxidant capacity, thereby reducing chondrocyte damage and slowing OA progression.

Among mitochondrial homeostasis-related interventions, oxidative stress is one of the earliest and most extensively studied targets of TCM-derived monomers. Representative studies on resveratrol have shown that it can reduce reactive oxygen species accumulation, improve mitochondrial polarization, and restore ATP production in chondrocytes under oxidative stress, thereby attenuating mitochondria-dependent apoptotic signaling. This suggests that the protective effects of certain polyphenolic compounds against OA extend beyond general anti-inflammatory mechanisms to directly target the core of mitochondrial function (Dave et al., 2008; Liang et al., 2014). Research on quercetin further extends this evidence chain to the three levels of “redox—energy metabolism—mitochondrial homeostasis”: on the one hand, quercetin can reduce ROS levels, increase GSH and GPx expression, restore mitochondrial membrane potential, oxygen consumption, and ATP levels, and increase mitochondrial copy number by activating the AMPK/SIRT1 pathway; on the other hand, in TBHP- or IL-1β-induced models, it can also alleviate oxidative stress-related apoptosis and inhibit the upregulation of catabolic factors such as MMP-3 and MMP-13, indicating that it possesses dual effects of improving mitochondrial energy supply and protecting the cartilage matrix (Hu et al., 2019; Zhang et al., 2020; Feng et al., 2019; Lv et al., 2022). Similarly, the role of curcumin is no longer limited to traditional anti-inflammatory effects; it can improve mitochondrial quality control via the AMPK/PINK1/Parkin axis in both *in vitro* and *in vivo* OA models, and can inhibit mitochondrial depolarization and caspase activation under oxidative/nitrosative stress conditions, thereby reducing oxidative damage while maintaining the continuity of mitochondrial energy conversion; Scutellarin, on the other hand, can alleviate IL-1β-induced ROS, lipid peroxidation, and matrix degradation by enhancing autophagy or mitophagy, increasing mitochondrial membrane potential, and activating the Nrf2 antioxidant system (Jin et al., 2022; Sadeghi et al., 2023). This suggests that flavonoids and polyphenolic monomers exhibit distinct common mechanisms in regulating mitochondrial oxidative stress (Marton et al., 2021; Chen et al., 2017; Liu et al., 2024; Liu et al., 2025) (Figure 2).

While the aforementioned studies placed greater emphasis on “mitigating oxidative damage,” recent research has further demonstrated that certain monomers derived from traditional Chinese medicine have begun to be used to remodel the mitochondrial energy metabolism network in chondrocytes (Zhang et al., 2023). Unlike earlier studies that merely observed a decrease in ROS, research on magnolol and dihydroquercetin has shifted the focus to the mitochondrial respiratory chain, deacetylation, and energy coupling: the former can improve mitochondrial respiratory chain function impaired by OA by activating SIRT3 and promoting COX4I2 deacetylation, thereby enhancing mitochondrial antioxidant defense and restoring chondrocyte metabolic balance; the latter also relies on SIRT3 to maintain mitochondrial homeostasis, improving the degenerative phenotype of chondrocytes while inhibiting mitochondrial apoptosis, suggesting that SIRT3 may serve as a crucial hub linking traditional Chinese medicine monomers to the remodeling of mitochondrial energy metabolism (Wang et al., 2018; Xia et al., 2023). In contrast, another class of active molecules represented by berberine and santalin exhibits more pronounced restorative effects on mitochondrial membrane potential and redox homeostasis: a sustained-release berberine system has been shown to increase mitochondrial membrane potential in chondrocytes stimulated by SNP and reduce the expression of caspase-3, ADAMTS-5, and MMP-13 expression in chondrocytes stimulated by SNP, while zitanqi can suppress mitochondrial and intracellular ROS production by activating Nrf2, thereby alleviating IL-1β-induced inflammatory mediator release and cartilage degeneration. This indicates that TCM-derived monomers can improve the “energy supply side” by enhancing the efficiency of the mitochondrial respiratory chain and stabilize the “damage side” by reducing the mitochondrial ROS burden; together constituting an important metabolic basis for delaying the progression of OA (Zhou et al., 2017; Xue et al., 2017) (Figure 2).

From a broader perspective, the regulation of mitochondrial oxidative stress and energy metabolism by TCM-derived compounds is not a pair of isolated processes, but rather a synergistic intervention centered on “maintaining mitochondrial functional continuity.” Among the currently studied natural compounds, several TCM-derived monomers have attracted particular attention because they are supported by relatively concentrated evidence and show clearer mechanistic relevance to mitochondrial homeostasis in OA. These representative compounds not only cover different chemical classes, but also reflect the main intervention patterns currently reported in this field. Recent studies have shown that isoquercetin can reduce ROS levels in aged chondrocytes, increase mitochondrial membrane potential, and inhibit SASP-related phenotypes by modulating the NOX4/Nrf2 redox imbalance, indicating that its regulation of mitochondrial redox balance has extended beyond simple antioxidant effects to the repair of age-related energy instability (Zhou et al., 2025). Other naturally occurring sulfur-containing active compounds, such as diallyl disulfide, have also been shown to alleviate IL-1β-induced chondrocyte damage by enhancing Nrf2 nuclear translocation and antioxidant enzyme expression, reducing lipid peroxidation, and inhibiting mitochondrial apoptosis signaling. This finding, combined with the aforementioned actions of resveratrol, quercetin, curcumin, baicalin, magnolol, and berberine, collectively demonstrating that: the value of TCM-derived monomers in OA lies not only in end-point phenotypes such as “anti-inflammation” or “inhibition of catabolism,” but also in their ability to reshape the metabolic resilience of chondrocytes at more upstream levels—including mitochondrial oxidative stress, maintenance of membrane potential, respiratory chain efficiency, and ATP supply—thereby providing sustained support for the subsequent restoration of matrix homeostasis and the delay of disease progression (Hosseinzadeh et al., 2017).

## 4 Compounds derived from traditional Chinese medicine protect chondrocytes by regulating mitochondrial dynamics and mitochondrial autophagy

In addition to mitochondrial oxidative stress and energy metabolism disorders, mitochondrial dysregulation is also a key mechanism underlying chondrocyte damage in OA, as it disrupts mitochondrial quality control, impairs cellular stress adaptation, and ultimately promotes apoptosis, senescence, and matrix degradation. Under normal conditions, mitochondria maintain morphological renewal, functional specialization, and stress adaptation through continuous fission and fusion (Chen L. et al., 2025; Tan et al., 2025; Lin et al., 2024). Key proteins such as DRP1 and FIS1 primarily participate in mitochondrial fission, while MFN1, MFN2, and OPA1 primarily mediate mitochondrial fusion; Once this balance is disrupted by inflammatory stimuli, aging, or abnormal mechanical loads, chondrocytes become prone to changes such as mitochondrial fragmentation, unstable membrane potential, decreased ATP production, and heightened responsiveness to external stimuli, ultimately driving apoptosis, senescence, and matrix degradation (Ansari et al., 2018; Cheung et al., 2024; Li et al., 2022). Recent studies have further demonstrated that mitochondrial dysregulation is not merely a parallel process to OA but is more likely a key mediator that amplifies pathological processes: for example, low SIRT3 expression can exacerbate mitochondrial respiratory dysfunction and is accompanied by impaired mitochondrial autophagy, whereas its restoration helps improve the clearance efficiency of damaged mitochondria and maintain chondrocyte homeostasis (Wang et al., 2018; Li et al., 2024; Hou et al., 2025). Against this backdrop, the value of TCM-derived compounds has gradually expanded from “anti-inflammatory” effects to “maintaining mitochondrial morphology and quality control networks.” Specifically, by regulating the fission-fusion balance, reducing excessive mitochondrial fragmentation, and restoring the renewal capacity of damaged mitochondria, these compounds enhance the metabolic resilience and survival of chondrocytes within the OA microenvironment (Shin et al., 2019; Lin et al., 2024; Xie et al., 2025; Jie et al., 2025; Deng et al., 2025).

To date, several single compounds derived from traditional Chinese medicine have been shown to improve OA phenotypes by modulating mitochondrial autophagy or mitochondrial dynamics; curcumin and baicalin are representative examples with relatively robust evidence. In both *in vitro* and *in vivo* OA models, curcumin enhances mitochondrial autophagy by activating the AMPK/PINK1/Parkin pathway, promoting the clearance of damaged mitochondria, and simultaneously reducing chondrocyte apoptosis, inflammatory responses, and matrix degradation. This indicates that its effects are not limited to simple antioxidant activity but involve direct intervention in mitochondrial quality control processes (Heidari et al., 2023; Zia et al., 2021). Baicalin also exhibits distinct mitochondrial-targeting characteristics. On the one hand, it can increase mitochondrial autophagy levels by inhibiting the PI3K/AKT/mTOR signaling pathway and activating PINK1/Parkin and PINK1/Drp1-related processes; while on the other hand, it can improve the decline in mitochondrial membrane potential as detected by JC-1 and downregulate apoptosis-related molecules such as Bax and cleaved caspase-3, suggesting it possesses dual advantages in restoring mitochondrial renewal and inhibiting mitochondrial-dependent damage (Wei et al., 2024; Huang et al., 2024). Furthermore, dihydroquercetin and its dual-responsive hydrogel system have been reported to inhibit chondrocyte degeneration by rebalancing the relationship between mitochondrial apoptosis and mitochondrial autophagy, with SIRT3 considered a key upstream regulator. This suggests that certain TCM monomers not only “enhance mitochondrial autophagy” but, more importantly, can reintegrate mitochondrial autophagy, membrane potential maintenance, and cell fate regulation into a relatively stable dynamic equilibrium (Zhang et al., 2024; Shao et al., 2024; D'Amico et al., 2022).

It is worth noting that the role of mitochondrial autophagy in OA is not simply a matter of “the more, the better”; rather, it is clearly stage-specific and requires moderation. Therefore, the true advantage of single compounds derived from traditional Chinese medicine lies not in mechanically upregulating a specific parameter, but in restoring the coordination between mitochondrial dynamics and mitochondrial autophagy. Previous studies have shown that abnormally activated Pink1-mediated mitochondrial autophagy may also be involved in the process of cartilage degeneration, whereas the moderate restoration of SIRT3-dependent mitochondrial autophagy and respiratory function can alleviate the OA phenotype. This suggests that precise rebalancing, rather than unidirectional promotion, is a more reasonable intervention strategy (He et al., 2024; Long et al., 2025). This has also been corroborated by recent studies on novel natural bioactive molecules. For example, acetylgingerone enhances mitochondrial autophagy by promoting the PINK1/Parkin signaling pathway, thereby inhibiting pyroptosis and slowing the progression of OA; while magnolol has demonstrated the potential to improve mitochondrial autophagy defects and restore mitochondrial function in SIRT3-related models; while some non-classical natural small molecules, such as β-hydroxybutyrate, α-ketoglutarate, and fasting-induced metabolic signaling, further suggest that maintaining the coupling of mitochondrial autophagy, dynamics, and metabolic functions is a key common mechanism for protecting chondrocytes and delaying the progression of OA (Cai et al., 2024; Mohammadi et al., 2024; Li et al., 2015). Therefore, based on the current evidence, research on the role of natural product-derived compounds from traditional Chinese medicine in regulating mitochondrial dynamics and autophagy has gradually shifted from describing correlations to reconstructing quality control networks. Its core significance lies in the sustained protection of chondrocytes and the slowing of articular cartilage degeneration through the clearance of damaged mitochondria, the reduction of mitochondrial fragmentation, and the maintenance of a functional mitochondrial population (Figure 2).

## 5 Monomers derived from traditional Chinese medicine delay the progression of OA by regulating mitochondria-related cell fate and matrix metabolism

**FIGURE 3:**
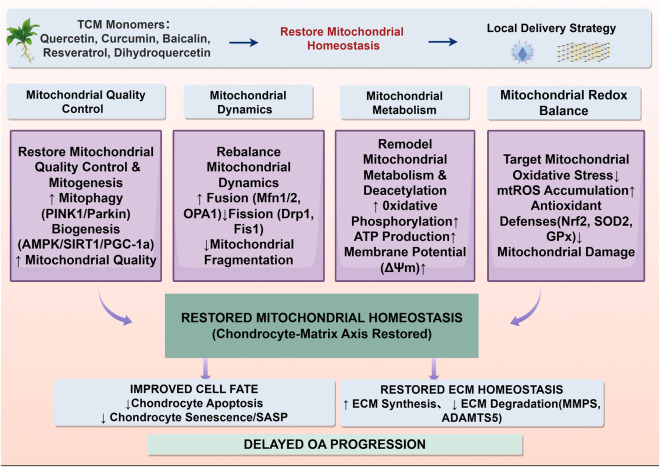
Schematic diagram illustrating the mechanism by which monomers derived from traditional Chinese medicine influence chondrocyte fate and matrix metabolism through the regulation of mitochondrial quality control. Monomers derived from traditional Chinese medicine can restore mitochondrial homeostasis in chondrocytes by improving mitochondrial quality control, reestablishing mitochondrial dynamic equilibrium, reshaping mitochondrial metabolism, and maintaining mitochondrial redox homeostasis. These mechanisms include promoting PINK1/Parkin-mediated mitochondrial autophagy and AMPK/SIRT1/PGC-1α-related biological processes, enhancing Mfn1/2 and OPA1-mediated fusion while inhibiting Drp1 and Fis1-related fission, improving oxidative phosphorylation, ATP production, and membrane potential, while simultaneously reducing mtROS accumulation and enhancing Nrf2/SOD2/GPx antioxidant defense. Ultimately, these effects help reduce chondrocyte apoptosis and senescence, restore the balance between ECM synthesis and degradation, and thereby delay the progression of OA.

Among the downstream consequences of mitochondrial homeostasis imbalance, disruption of chondrocyte fate is one of the most direct and critical events. This is mainly reflected by increased apoptosis, accelerated senescence, and sustained activation of inflammation-related injury pathways, which ultimately converge in the breakdown of extracellular matrix homeostasis (Buhrmann et al., 2017). Previous studies have shown that resveratrol can alleviate oxidative stress-induced chondrocyte apoptosis by improving mitochondrial polarization and ATP production, suggesting that its regulation of cell fate goes beyond superficial anti-inflammatory effects and is closely related to the restoration of mitochondrial function (Im et al., 2012). Quercetin, on the other hand, exhibits a more comprehensive protective cascade in various OA models (Song and Yu, 2024). It inhibits endoplasmic reticulum stress and mitochondria-dependent apoptosis through AMPK/SIRT1-related mechanisms, while simultaneously reducing ROS (ROS) levels, maintaining mitochondrial membrane potential, and further suppressing the expression of catabolic factors such as MMP-3 and MMP-13, thereby linking mitochondrial protection, cell survival, and matrix stability (Liao et al., 2024; Wang et al., 2023). Similarly, natural monomers such as baicalin, vacarinin, and curcumin have been shown to alleviate IL-1β-or SNP-induced mitochondria-related apoptosis by inhibiting Bax upregulation, cytochrome c release, and caspase-9/caspase-3 activation, accompanied by the restoration of Aggrecan and COL2A1 expression and downregulation of ADAMTS5 and MMP13, indicating that the regulation of mitochondrial-related cell fate by monomers derived from traditional Chinese medicine ultimately manifests as the restoration of the balance between chondral matrix synthesis and degradation (Huang et al., 2021; Pan et al., 2017; Cao et al., 2018; Yang et al., 2018) (Figure 3).

In addition to apoptosis, mitochondrial dysfunction is closely associated with chondrocyte senescence; the continuous accumulation of senescent cells amplifies inflammatory responses and promotes matrix degradation through the secretory phenotype of the senescent-associated secretory phenotype (SASP). Therefore, “reducing the senescence burden” is emerging as a key criterion for evaluating whether single compounds derived from traditional Chinese medicine possess disease-modifying potential (Li et al., 2026). Previous studies have shown that isoquercetin can reduce ROS levels in senescent chondrocytes, increase mitochondrial membrane potential, and inhibit the expression of senescence-associated molecules such as p16 and p21 by regulating the NOX4/Nrf2 redox imbalance, thereby alleviating SASP-related inflammation and matrix degeneration (Yu et al., 2024). Natural bioactive molecules such as ginkgolide K, hyperoside, and senkyunolide I have demonstrated similar effects; they can inhibit inflammation and apoptosis, alleviate oxidative stress, and simultaneously correct matrix metabolic abnormalities—such as the downregulation of COL2A1 and aggrecan and the upregulation of MMP13 and ADAMTS5—in various models. Some studies have further attributed these mechanisms to Nrf2/HO-1 activation, inhibition of NF-κB, and mitigation of mitochondrial-related stress (Liu R. et al., 2023; Sun et al., 2023). Notably, recent studies no longer settle for descriptions such as “a certain compound inhibits apoptosis or reduces MMP13,” but instead emphasize whether these phenotypic improvements are based on the reversal of mitochondrial-related pathological processes; for example, urostatin A not only improves mitochondrial health and respiratory function but also alleviates cartilage degeneration, synovitis, and pain (Li et al., 2025; Han et al., 2024). This suggests, at a higher level, that maintaining the homeostasis of mitochondrial-related cell fate may be a crucial prerequisite for natural active compounds to achieve sustained chondroprotective effects (Chen H. et al., 2025; He et al., 2021).

From the perspective of matrix metabolism, the true translational value of TCM-derived monomers in regulating mitochondrial-related cell fate lies not in their ability to merely “protect a single cell” in isolation, but rather in their capacity to continuously restore the synthesis-degradation balance of articular cartilage by improving chondrocyte survival, senescence, and inflammatory responses. Studies on natural monomers such as Aucubin, Hederagenin, and Periplogenin have shown that when mitochondrial-related apoptosis and inflammatory pathways are inhibited, synthetic phenotypes in chondrocytes—including COL2A1, Aggrecan, and SOX9—are restored, while degradative or inflammatory mediators such as MMP13, ADAMTS5, iNOS, and COX-2 are significantly downregulated (Wang et al., 2019; Zhou et al., 2026). This suggests that improved matrix metabolism is not an isolated event but rather an inevitable outcome of the recalibration of mitochondrial-related cell fate (Young et al., 2017). On the other hand, recent studies have also shown that individual compounds such as puerarin have the ability to simultaneously inhibit apoptosis, promote matrix synthesis, and reverse the IL-1β-induced degradation phenotype, suggesting that certain natural compounds may possess a combined potential for mitochondrial protection, anti-inflammation, and promotion of matrix repair (Zhang Y. et al., 2025). Therefore, based on current evidence, the core mechanism by which monomers derived from traditional Chinese medicine delay the progression of OA through the regulation of mitochondrial-related cell fate does not lie in the improvement of a single pathway or a single indicator, but rather in their ability to interrupt the continuous pathological chain—comprising mitochondrial damage, exacerbated apoptosis or senescence, amplified inflammation, and matrix degradation—at its upstream origin, ultimately contributing to the long-term maintenance of cartilage homeostasis; This also provides a more solid theoretical foundation for the future shift from interventions targeting mitochondrial homeostasis to disease-modifying interventions (Wu et al., 2023; Chang et al., 2024) (Figure 3).

## 6 Conclusion and outlook

Overall, mitochondrial homeostasis imbalance is no longer merely a concomitant phenomenon in the pathological process of OA; rather, it should be regarded as a key hub linking mechanical stress, oxidative damage, inflammatory amplification, dysregulated cell fate, and matrix metabolic disorders. Research based on this understanding indicates that the therapeutic value of TCM-derived monomers for OA has gradually shifted from the early, general concepts of “anti-inflammatory and antioxidant” effects to a more mechanism-driven approach of “mitochondria-targeted regulation.” Based on current evidence, these active monomers can sustainably improve the metabolic resilience and survival of chondrocytes within the OA microenvironment by alleviating mitochondrial oxidative stress, restoring membrane potential and ATP production, maintaining mitochondrial homeostasis, promoting moderate mitochondrial autophagy, and inhibiting mitochondrial-associated apoptosis and senescence. Furthermore, these upstream effects ultimately translate into downregulation of inflammatory factors, inhibition of catabolic molecules such as MMP13 and ADAMTS5, and restoration of matrix synthesis phenotypes involving COL2A1 and Aggrecan. This establishes a relatively complete chain of action: “mitochondrial protection—improved cell fate—reconstruction of matrix homeostasis—delayed disease progression.” It is precisely for this reason that mitochondrial homeostasis provides a more unified and explanatory theoretical framework for reintegrating the multi-target effects of TCM-derived monomers in OA.

However, it must also be acknowledged that current research in this field still faces significant limitations. First, the vast majority of evidence continues to come primarily from *in vitro* chondrocyte experiments and rodent models; high-quality studies that can truly demonstrate the consistent therapeutic efficacy and clear targeted effects of TCM-derived monomers at the local joint site in OA remain limited. Second, although many studies have observed phenomena such as decreased ROS levels, restored membrane potential, enhanced autophagy, or reduced apoptosis, the causal chain remains insufficiently robust. These findings often remain at the level of “improved correlations,” lacking experiments involving mitochondrial-specific blockade, gene editing, or functional rescue to demonstrate that mitochondrial homeostasis is indeed a key mechanism. Furthermore, there remain discrepancies in how different studies interpret the same biological processes, particularly regarding mitochondrial autophagy and kinetic regulation. Benefits are not simply achieved through enhancement or inhibition; the truly effective approach is likely to restore dynamic equilibrium—a question that many current studies have yet to fully address. Furthermore, systematic comparisons of the potency, target specificity, pharmacokinetic profiles, bioavailability, and delivery efficiency to joint tissues among different TCM-derived monomers are still lacking. This leaves the critical question of “which monomers are most worthy of further development” unanswered. In other words, while this field has established preliminary evidence of “effectiveness,” there remains a significant gap in understanding “why it works, which compounds are superior, and how to translate these findings into clinical applications.”

Future research should focus on three key areas. First, mechanistic studies should move beyond phenotype description toward causal validation using mitochondrial tracing, mitochondria-specific interventions, multi-omics integration, and genetic approaches. Second, research should expand from isolated chondrocytes to the whole-joint microenvironment, with greater attention to the interactions among synovial cells, macrophages, subchondral osteocytes, sensory neurons, and mitochondrial homeostasis. Since OA is essentially a disease affecting the entire joint, a chondrocyte-centric explanation alone is no longer sufficient to capture the full picture of the disease. Third, at the translational level, greater emphasis should be placed on the pharmaceutical development of TCM-derived monomers and the optimization of delivery strategies. For example, by utilizing nanocarriers, sustained-release hydrogels, intra-articular targeted delivery, or structural modifications to enhance their stability, local accumulation capacity, and long-term safety, we can advance these compounds from laboratory phenomena to disease-modifying drug candidates with practical application potential. Currently, the translation of this field into clinical practice still faces multiple obstacles, including the low bioavailability of certain active ingredients derived from traditional Chinese medicine, limited efficiency of local delivery to joints, and insufficient evaluation of long-term safety. At the same time, there remains a lack of evidence based on human samples and high-quality clinical studies, which also limits the transition from experimental research to practical application. Overall, targeting mitochondrial homeostasis offers a more promising direction for TCM-derived monomers in the treatment of OA than traditional “anti-inflammatory and analgesic” approaches. The true value of this direction lies not in adding a few more “effective monomers” to the list, but in shifting the treatment paradigm for OA from controlling end-stage symptoms to reshaping the pathological core.
